# Primary Human Placental Trophoblasts are Permissive for Zika Virus (ZIKV) Replication

**DOI:** 10.1038/srep41389

**Published:** 2017-01-27

**Authors:** Kjersti M. Aagaard, Anismrita Lahon, Melissa A. Suter, Ravi P. Arya, Maxim D. Seferovic, Megan B. Vogt, Min Hu, Fabio Stossi, Michael A. Mancini, R. Alan Harris, Maike Kahr, Catherine Eppes, Martha Rac, Michael A. Belfort, Chun Shik Park, Daniel Lacorazza, Rebecca Rico-Hesse

**Affiliations:** 1 Departments of Obstetrics & Gynecology, Division of Maternal-Fetal Medicine at Baylor College of Medicine & Texas Children’s Hospital, Houston, TX, USA; 2 Department of Molecular & Human Genetics at Baylor College of Medicine, Houston, TX, USA; 3 Department of Molecular & Cellular Biology at Baylor College of Medicine, Houston, TX, USA; 4 National School for Tropical Medicine at Baylor College of Medicine, Houston, TX, USA; 5 Department of Molecular Virology & Microbiology, Baylor College of Medicine, Houston, TX, USA; 6 Integrative Molecular and Biological Science Program, Baylor College of Medicine, Houston, TX, USA; 7 Department of Pathology & Immunology, Baylor College of Medicine & Texas Children’s Hospital, Houston, TX, USA.

## Abstract

Zika virus (ZIKV) is an emerging mosquito-borne (*Aedes* genus) arbovirus of the *Flaviviridae* family. Although ZIKV has been predominately associated with a mild or asymptomatic dengue-like disease, its appearance in the Americas has been accompanied by a multi-fold increase in reported incidence of fetal microcephaly and brain malformations. The source and mode of vertical transmission from mother to fetus is presumptively transplacental, although a causal link explaining the interval delay between maternal symptoms and observed fetal malformations following infection has been missing. In this study, we show that primary human placental trophoblasts from non-exposed donors (*n* = 20) can be infected by primary passage ZIKV-FLR isolate, and uniquely allowed for ZIKV viral RNA replication when compared to dengue virus (DENV). Consistent with their being permissive for ZIKV infection, primary trophoblasts expressed multiple putative ZIKV cell entry receptors, and cellular function and differentiation were preserved. These findings suggest that ZIKV-FLR strain can replicate in human placental trophoblasts without host cell destruction, thereby serving as a likely permissive reservoir and portal of fetal transmission with risk of latent microcephaly and malformations.

Human infection with ZIKV, an emerging mosquito-borne flavivirus (*Flaviviridae* family, *Flavivirus* genus) that is closely related to the Spondweni serocomplex, has reached pandemic levels in the Americas with at least 32 countries or territories reporting infection over the interval from May, 2015 through June, 2016 (http://www.cdc.gov/zika/geo/index.html). Previous outbreaks of ZIKV were largely sporadic across Southeast Asia and equatorial African belts, but later spread east resulting in an outbreak in Yap Island in 2007, followed by epidemics in French Polynesia, New Caledonia, the Cook Islands, and Easter Island in 2013 and 2014^1^,^2^.

Until recently Zika viral illness was thought to be self-limiting and resembling that of dengue and chikungunya with clinical manifestations of fever, headache, arthralgia, myalgia and maculopapular rash. Perinatal transmission of ZIKV and Guillain-Barre syndrome associated with ZIKV were thought to be relatively limited based on incidence rates in the French Polynesia outbreak (2 and 73 instances respectively out of 28,000 cases)^1^,^3^. However, as ZIKV spread to the Americas (reaching 1.3 million autochthonous cases by December 2015), an approximate 20-fold increase in congenital cases of microcephaly with brain and ocular malformations was reported throughout northeast and southeast Brazil^4^,^5^. Although no other flavivirus is known to cause disseminated fetal neural malformations in humans, worldwide concern for latent viral disease was raised following several case reports demonstrating ZIKV RNA in the amniotic fluid, placenta, and fetal neural tissue weeks to months after initial maternal infection^3^,^4^,^5^,^6^,^7^,^8^. More recently, a prospective cohort of 88 symptomatic gravidae from Rio de Janeiro were followed throughout gestation^9^. In this preliminary report, 42 of the 72 symptomatic gravidae who tested positive for ZIKV underwent ultrasound examination, with 29% (12 of 42 ZIKV) demonstrating variable findings on ultrasound, ranging in presumptive severity from CNS lesions with microcephaly, to isolated findings suggestive of placental insufficiency such as fetal growth restriction, or abnormal umbilical artery Doppler velocimetry or amniotic fluid volumes^9^. To date, neither a reservoir permissive for ZIKV replication nor a potential portal establishing a causal route for evident fetal neurotropism or placental insufficiency has been well-established in humans. One obvious conduit would be the placenta itself, with the differentiated placental trophoblast cells (*i.e.,* cytotrophoblasts and syncytiotrophoblasts) potentially serving as reservoirs for viral infection and replication.

The placenta consists of early differentiated and highly specialized epithelial cells (trophoblasts) and blood vessels in a branched network of supportive connective tissue (recently reviewed in ref. 10). The essential building blocks of the placenta are the chorionic villi, whose formation occurs with differentiation of the multi-potent trophoblast progenitors, the cytotrophoblasts (CTB). CTB detach from the trophoblast basement membrane surrounding the chorionic villi, and fuse to form a continuous layer of terminally differentiated syncytiotrophoblasts covering the villous surface. These villi both float in maternal blood as villous syncytiotrophoblasts, and form columns of non-polarized cells (extravillous trophoblasts), some of which endovascularly invade the uterine wall to anchor the placenta to the uterus and divert maternal blood to the intervillous space^10^. This intimate maternal-placental-fetal connection enables exchange of nutrients and waste, alongside production of hormones and extracellular vesicles, the latter of which is reported to largely protect against vertical transmission of viruses^10^,^11^,^12^.

Of interest to the current report, Bayer *et al*.^11^ employed high passage ZIKV strains from remote outbreaks (African strain MR766 and Asian strain FSS13025) and attempted to infect primary human trophoblasts at term. They observed that these historic and non-contemporaneous strains could infect placental trophoblast cell lines, but not primary human trophoblasts. Of note, their measure of infectivity was the presence of the negative viral RNA strand at early times following infection (24 and 48 hours post infection), and all replication comparisons were relative to human brain microvascular endothelial cells^11^. They further observed that conditioned media from human trophoblasts infected with these serially passaged ZIKV from the African (Ugandan) and Asian (Cambodian) outbreaks presumptively contained Type III interferon (IFNλ1), which potentially enables downstream expression of interferon stimulated genes that could restrict virus replication^11^. A more recent study from Quicke *et al*. demonstrated that human placental macrophages, or Hofbauer cells, are susceptible to infection by a recent Puerto Rican strain of ZIKV (PR 2015)^12^. Moreover, in contrast to the observations of Bayer and colleagues, Quicke *et al*. reported that CTBs were capable of productive ZIKV replication when trophoblasts were propagated longer (72 to 96 hours) in culture. In addition, they did not detect the presence of IFNλ1 in their infected trophoblast cultures, and attributed the discordance between their study and that of Bayer *et al*. to differences in time points assessed and preferential use of contemporaneous rather than distal, high passage historical viral strains^12^.

Despite the putative role of the placenta in modulating risk of fetal infection, not all viruses are inhibited in their passage through the placenta, and maternal to fetal vertical transmission is well documented^13^,^14^. However, viral infection and replication in placental cells themselves appears to be much less common. Among the *Flaviviridae*, only hepatitis C is known to undergo limited permissive replication in primary human term syncitialized cytotrophoblasts, but does not cause congenital malformations^13^. Cytomegalovirus (CMV; family *Herpesviridae*) demonstrates focal infection in the CTB progenitor cells of floating villi, with productive replication and cell-cell virus spread in interstitial invasive cytotrophoblasts^14^. CMV is the leading cause of congenital viral infections, and manifests clinically as microcephaly and fetal growth restriction with intracranial calcifications, accompanied by varying susceptibility and occurrence of seizures, developmental delay, and congenital retinitis and (most commonly) sensorineural hearing loss^14^. Generally, it is assumed that viral-mediated fetal microcephaly with malformations is a result of proliferative or maturational defects of neurons with death of cortical progenitor cells. Indeed, ZIKV has recently been observed to infect and to attenuate human neural progenitor cell (hNPC) growth and differentiation *in vitro*, and hNPC derived from induced pluripotent stem cells release ZIKV infectious particles^15^.

In recent weeks, other investigators working in relevant murine models have reported initial findings related to the potential for ZIKV trans-placental passage and subsequent vertical transmission. In the first of two elegant experiments in mice, Miner *et al*.^16^ infected fetal mice heterozygous for Type I interferon signaling defects (*Ifnar1*^+/−^) with ZIKV strain H/PF/2013 (French Polynesia, 2013) which had been serially passaged and propagated in Vero cells (African green monkey kidney epithelial cells). Fetal infectivity was achieved by inoculating dams (*Ifnar1*^−/−^) on embryonic days 6.5 or 7.5 (E6.5 or E7.5), and a high rate of pregnancy loss with fetal ocular and brain viral pathogenesis was subsequently observed. Of note, fetal loss was accompanied by a 1000-fold observed elevation in placental viral RNA quantification relative to maternal serum, alongside direct visualization of placental trophoblast and endothelial cell infection^16^. Similarly, Cugola *et al*.^17^ utilized a more recent Americas strain (ZIKV^BR^, isolated from a viremic subject in northeastern Brazil in 2015. The strain was alternately propagated in *A. albopictus* mosquito cells (C6/36), but with limited passage and subculturing. In an equally elegant set of experiments, C57BL/6 or SJL pregnant mice were infected in mid-gestation (E10–13), and subsequent fetal growth restriction alongside brain and ocular malformations in the pups were observed. Accompanying these fetal malformations were abundant ZIKV^BR^ RNA particles in multiple fetal tissues, with highest recovery in the fetal brain, followed by the spleen, kidney, and liver; however, placental expression was not examined^17^. Taken together, these findings by other investigators provide direct evidence for genetically or immunologically manipulated murine placental trophoblasts being permissive for infection^16^ or indirect evidence of transplacental passage^17^ from mother to fetus.

## Study rationale

In the current study, we reasoned that the recent murine models^16^,^17^, epidemiology^3^,^4^,^5^ and clinical presentation^4^,^5^,^6^,^7^,^8^,^9^ of ZIKV infection in pregnancy are most consistent with transfer of virus from mother to fetus during gestation, and that the temporal presentation of available cases suggests that the placenta may be a portal and permissive for infection and replication. In order to best recapitulate the current emerging pandemic, we aimed to determine if non-proliferating primary human trophoblasts (largely comprised of trophoblasts differentiated *in vitro* to syncitiotrophoblasts) isolated from a significant number (n = 20) non-infected, non-exposed, and unrelated subjects in the third trimester could be infected by a single passage recent epidemic ZIKV isolate from a viremic but non-pregnant human subject.

## Results

As recently described^18^, ZIKV-FLR was isolated by inoculating *A. albopictus* C6/36 mosquito cells with serum from a non-pregnant subject infected over a short interval stay in Barranquilla, Colombia (Supplementary Materials; ref. 18). The subject’s serum was negative by qRT-PCR for dengue (DENV), Chikungunya (CHIKV), but positive for ZIKV RNA using previously described methods^18^,^19^,^20^. We used virus from only one cell culture passage as input for all infection experiments, to minimize cell culture adaptation, and to reflect a virus population that would be initially infecting maternal blood, with later spread to the fetus. We compared the replication of our FLR clinical isolate of Zika virus to that of a dengue serotype 2 strain virus (strain K0049, SE Asian genotype); DENV serotype 2 grows at very high rates in human target cells, but has not been shown to cause fetal malformations after billions of human infections around the globe^21^,^22^. The number of tissue culture infectious doses (TCID) applied to each trophoblast culture well, for both ZIKV and DENV viruses (ZIKV: 1 × 10^5^ RNA copies were equal to 10 TCIDs, DENV: 1 × 10^5^ RNA copies were equal to 100 TCIDs) were quantitated by inoculating Vero cells with limiting virus dilutions and testing for viral protein expression, utilizing a monoclonal antibody (via IFA)^18^.

**Figure 1:**
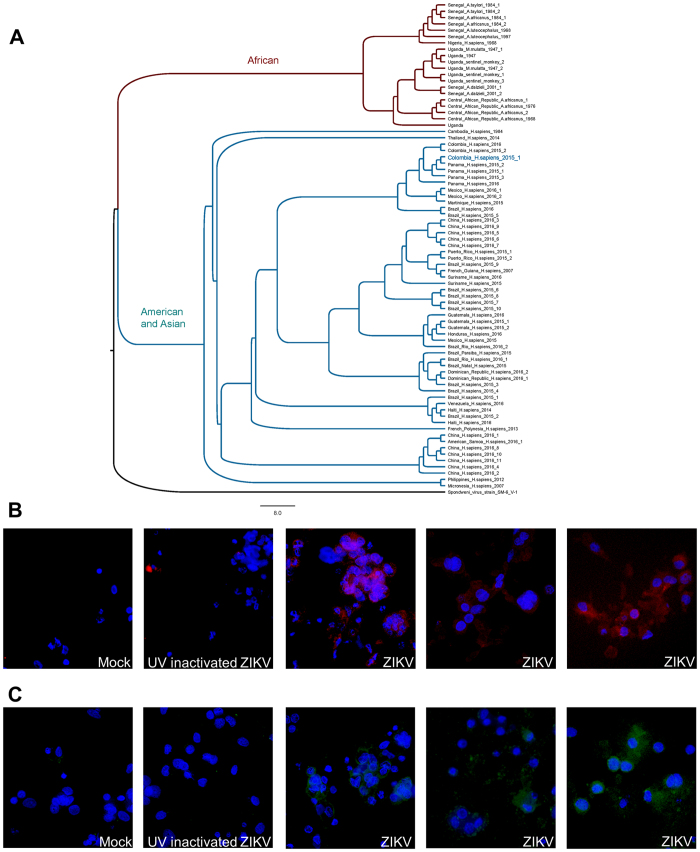
Phylogenetic characterization of ZIKV-FLR and demonstration of its replication in primary human trophoblasts. (**A**) Phylogenetic tree of full genome nucleotide sequences of all 77 currently available ZIKV strain complete genome or polyprotein CDS assemblies (accessed June, 2016); ZIKV-FLR strain is shown bolded in blue and annotated as Colombia_H_sapiens_2015_1. Sequences were aligned using MAFFT v7.0 (Katoh; ref. 23) with the accurate (L-INS-i) setting. PAUP* v4.0 (Swofford; ref. 24) automated model selection was used to identify optimal likelihood parameters based on the best AICc. A Neighbor Joining tree was constructed and the tree was visualized using FigTree v1.4.2 (Rambaut; http://tree.bio.ed.ac.uk/software/figtree/). Leaf nodes were labelled as country_host_year of collection. Methods and accession numbers are provided in Supplementary Materials. In panels B and C, primary human trophoblasts infected with ZIKV-FLR strain were analyzed at 5 days post-infection for the presence of viral replicative complexes (**B**) and envelope glycoprotein expression (**C**) by immunofluorescence, in three different unrelated placental donors. (**B**) Viral dsRNA was detected in primary human trophoblasts using the J2 monoclonal antibody and AF568-conjugated secondary anti-mouse IgG; the J2 antibody is not specific for ZIKV, but only detects viral replicative complex. Both mock-infected trophoblasts and those infected with UV-inactivated 1 × 10^5^ ZIKV (left two panels) failed to demonstrate evidence of dsRNA viral replicative complexes (red labeling). By contrast, presence of replicative viral complexes were observed in primary human trophoblast cultures on day 5 post infection among donors infected with 1 × 10^5^ RNA copies of ZIKV (red labeling on right three panels, with three separate unrelated donor trophoblasts). (**C**) Viral E-glycoprotein expression, using the 4G2 monoclonal antibody and FITC-conjugated secondary anti-mouse IgG (green labeling) in parallel cultures to panel B: mock-infected cells, UV-inactivated 1 × 10^5^ ZIKV, and 1 × 10^5^ ZIKV in three separate unrelated donor trophoblasts.

Phylogenetic trees were generated from all currently available complete ZIKV sequences (*n* of 77; Fig. 1A). The resultant phylogenetic tree demonstrates that ZIKV strain FLR (GenBank accession KU820897) was most similar to other reported ZIKV strains from the recent Americas pandemic, with evident phylogenetic delineation noted between African strains and the current Americas and recent Asian strains (Fig. 1A)^23^. This strain-level clustering may be of potential importance, given both the absence of reports regarding perinatal infection and fetal malformations prior to the French Polynesian outbreak and the previous report of Bayer *et al*.^11^.

**Figure 2:**
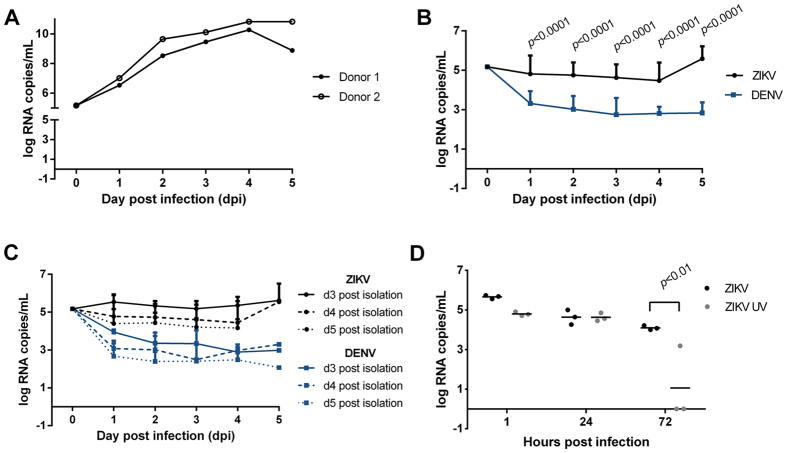
Quantitative data demonstrating primary human placental trophoblasts are permissive for ZIKV infection and replication, but not DENV infection nor replication. (**A**) In the initial two experiments, trophoblasts were isolated from two unrelated donors (donor 1, donor 2) and plated at 1 × 10^6^ cells/well. On day 5 (donor 1) or day 4 (donor 2) after isolation, now differentiated trophoblasts were infected with ZIKV FLR. (**B**) Zika and dengue virus growth curves in primary trophoblasts, after infection with 1 × 10^5^ RNA copies per virus. All wells contained 1 × 10^6^ cells, except for one donor, which contained 1 × 10^5^ cells. The average of 16 ZIKV and 12 DENV infections are projected (see Fig. S1). (**C**) Zika and dengue viral growth curves, by day of trophoblast cell infection post isolation (i.e., *ex vivo* culture). No differences with ZIKV inoculated cultures were observed 3 days after trophoblast differentiation had occurred (comparing 3 (solid lines), 4 (dashed lines) or 5 (dotted lines) days post isolation). (**D**) ZIKV infection is dependent upon active viral replicons, as UV treated ZIKV RNA was not detectable 72 hours post infection (*p* < 0.01, ANOVA with Bonferroni post-hoc).

Primary human trophoblasts were permissive to infection with single passage, non-culture adapted isolate ZIKV-FLR (Fig. 1B, C and Fig. 2). Qualitative evidence of both infection and active replication was obtained using immunofluorescence (IF) labeling. As shown in Fig. 1B and C, first passage ZIKV was incubated for 1 hour with primary human trophoblasts seeded at 1 × 10^6^ placental cells/well on day 4 following isolation. For control, trophoblast cultures either mock or infected with UV inactivated (irradiated) ZIKV, at an equivalent initial input, were similarly subjected to IF. Detection of dsRNA virion replicative complexes was made with the J2 monoclonal antibody^25^, and the E glycoprotein was detected by 4G2 monoclonal antibody labeling^26^. Following initial infection for 1 hour, the inoculum was then removed, and trophoblasts were gently washed with culture medium then continuously incubated at 37 °C and 5% CO2 over the experimental window (up to 5 days). Qualitative evidence consistent with active replicating virus, as detected by J2 labeling of dsRNA, was observed (red labeling, Fig. 1B), as was the presence of E glycoprotein (green labeling, Fig. 1C). By contrast, neither mock infected nor UV irradiated ZIKV demonstrated active replication in primary human trophoblasts (left panels, Fig. 1B and C). Of note, and as stated above, neither the 4G2 monoclonal antibody nor J2 labeling is specific for ZIKV replication.

Therefore, for quantitation of ZIKV-FLR replication in primary human trophoblasts, direct measures of ZIKV specific RNA transcript was performed. In the initial two experiments, first passage ZIKV at an input of 1 × 10^5^ RNA copies/ml (10 TCID) was incubated for 1 hour with primary human trophoblasts seeded at 1 × 10^6^ placental cells/well on day 5 (donor 1) or day 4 (donor 2) following isolation. On subsequent days post infection (dpi), ZIKV RNA expression was measured with observation of multi-log fold (from 1 × 10^5^ to 1 × 10^11^) increases in RNA copies/ml observed from 1 to 5 dpi (Fig 2A). We thereafter repeated the experiment in a subsequent 18 unrelated placental donors using a later passage viral stock (Table S2), and found that the same viral RNA input of ZIKV and DENV led to very distinct growth curves, with dengue viral RNA decreasing rapidly over the course of 3 days, while that of Zika did not (Fig. 2B and C; Fig. S1). This was not secondary to slower decay of ZIKV, since UV irradiated ZIKV decayed rapidly with no RNA viral particles detected by 72 hours post infection (Fig. 2D). We consistently observed that while the fold increase in ZIKV replication varied from one donor to the next, in all instances primary human trophoblasts were more permissive for ZIKV compared with that of DENV (where comparisons were made, *p* < 0.0001; Fig. 2B, Fig. S1), regardless of the numbers of days post trophoblast isolation or dpi (Fig. 2C; day 2, 3, 4 or 5 post isolation serving as day of infection, with representative βhCG plateau data shown in Fig. S2). ZIKV innoculation did not result in trophoblast dysfunction nor senescence nor death, as evidenced by ongoing elevations in βhCG production (Fig. S2). Consistent with the cultures containing a high purity population of placental trophoblasts, largely syncitialized and differentiated by day 3 in culture (as evidenced by plateaued βhCG production; Fig. S2), there was no evidence of peripheral mononuclear cells, lymphocytes, nor dendritic cells by either sequential flow cytometry analysis (Fig. S3) nor by inflammatory cytokine production after ZIKV inoculation (Fig. S4).

**Figure 3:**
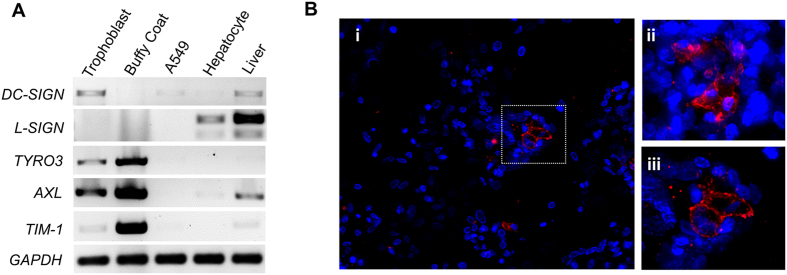
Putative ZIKV cell entry receptors are expressed in syncytialized primary human trophoblasts. (**A**) Select viral receptor targets were measured on day 5 post isolation. Cultured primary trophoblasts exhibit *AXL, DC-SIGN, TYRO3* and *TIM-1*, but not *L-SIGN* transcription as assessed by RT-PCR detection of human transcript message. Isolated reverse transcribed mRNA was amplified using intron/exon junction spanning primers with a GAPDH as a loading control. For DC SIGN and L- SIGN, primers were designed to capture the multiple transcript variants. Discovered major bands are shown comprising RefSeq transcript variant 1 for DC-SIGN, and transcript variants 1, 8, 9 and 10 (larger band), and variants 7 and 11 (smaller band) for L-SIGN. Isolated buffy coat, A549 cells, isolated primary human hepatocytes and whole human liver were used as controls. (**B**) Immunofluorescence labeling for the expression and cellular localization of the Axl presumptive ZIKV entry receptor. Representative images of day 5 uninfected trophoblasts were imaged following antibody probing with goat polyclonal antibody and Alexa Fluor^®^ 647 labeled anti-goat secondary antibody for detection (i). Z-stacks of 20X immunofluorescence images were deconvolved and maximum projected. Areas were abstracted (ii-iii) to visualize cellular localization at the cell membrane. Panel iii is indicated by the boxed area.

While multiple human surface proteins likely facilitate ZIKV entry into cells, the precise viral receptor(s) and mechanisms of entry remain unknown^14^,^15^,^16^,^17^. Several of these proteins are sufficient to support ZIKV entry into transfected HEK293 cells^17^ with low infectivity, including DC-SIGN, TIM1, TYRO3, and AXL. Based on our observed permissiveness for ZIKV infection and replication, we hypothesized that uninfected human placental trophoblasts may express receptor transcript and protein during their process of differentiation to cytotrophoblasts and syncytiotrophoblasts. Consistent with viral entry and permissiveness for infection, we observed the presence of four of the putative ZIKV cell entry receptor transcripts in primary human trophoblasts differentiated in culture (Fig. 3A), with cell surface membrane localization of AXL (Fig. 3B) in a smaller subpopulation of trophoblasts by immunoflourescence. Taken together, these findings are further support that our observed permissiveness to ZIKV FLR infection in trophoblasts cannot be attributed to either low level mononuclear or dendritic cellular contamination, or to cytolysis of early inoculated cells.

**Figure 4:**
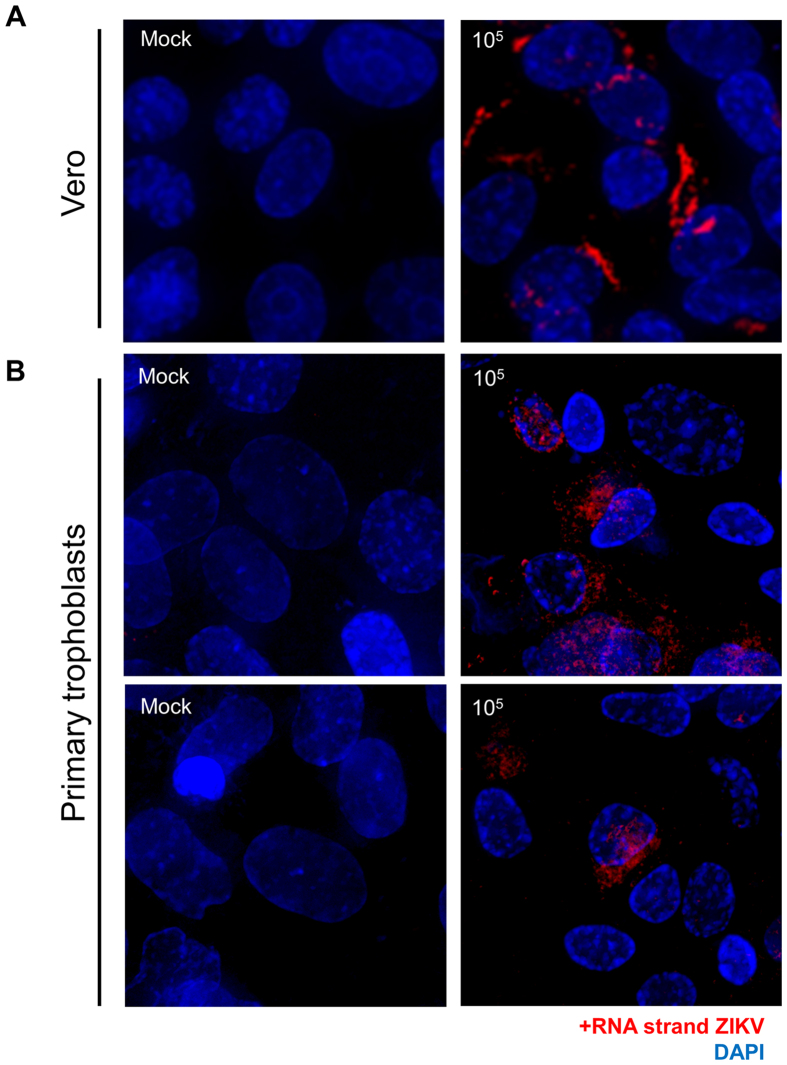
Direct visualization of ZIKV viral RNA in primary human placental trophoblasts by single molecule RNA FISH. (**A**) Vero cells mock (left) or infected with 10^5^ RNA copies with ZIKV-FLR strain (right) were probed with specific fluorescently-labeled oligo panels to ZIKV RNA at 5 dpi to demonstrate the specificity of the RNA FISH probe set (red). (**B**) Primary human placental trophoblasts on post-isolation day 4 were also mock (left panels) or infected with ZIKV-FLR strain (right) at 1 × 10^5^ RNA copies, and were fixed at 5 dpi. Images on the right demonstrate the positive strand of ZIKV in infected cells compared to mock controls (left). Select images from six separate experiments captured at 60X/1.42 NA, deconvolved and maximum projected are shown. All staining in both Vero and trophoblast cultures is cytoplasmic, with appearance of overlay reflective of dimensionality.

In order to visualize single molecules of ZIKV viral RNA for both the positive strand (+strand) and the actively synthesized negative strand (−strand) with cellular localization *in situ*, we developed and synthesized two sets of individually fluorophore-labeled probe sets (Stellaris™, LGC Biosearch Technologies) for use in single molecule RNA fluorescence *in situ* hybridization (FISH) coupled with deconvolution microscopy. The + strand probe set was generated against KU365780.1, nts 125–8011 (designed to detect the ZIKV positive polyprotein coding RNA strand *in situ*), spanning the consensus region. The –strand probe set was designed against the reverse complement of KU365780.1, nts c8011–125 (designed to detect the ZIKV negative replication template RNA strand *in situ*) spanning the complement of the consensus region as described in Supplemental Methods. Each oligo to the positive strand was labeled with one Quasar^®^ 570 dye (a cyanine 3 analog) at the 3′ end; the negative viral strand probe set was 3′-labeled with ATTO 647 N, and further purified to remove unreacted dye. Optimization of probe and detection was performed with mock or ZIKV-FLR infected Vero African green monkey kidney cells (an established cell line susceptible to certain viral infections; Fig. 4A), and comparison was thereafter made to DENV (data not shown) or ZIKV inoculated primary placental trophoblasts (Fig. 4B). Subcellular localization was relative to DAPI (nuclear labeling), and cytoplasmic localization was appreciated using wide-field microscopy (60X/1.42 NA) and deconvolution (Fig. 4 and Fig. S5). Our minimal threshold level of detection of viral RNA by FISH in Vero cells was estimated to approximate 1 × 10^2^ viral RNA particles/ml, and our detection in trophoblasts met this level of detection (Fig. S5). As anticipated, Vero cell monolayers labeled positive for cytoplasmic ZIKV on both the + and − strand following infection with the FLR strain for up to 5 days, but not with mock (Fig. 4A; Fig. S5) nor DENV (data not shown). Although primary trophoblasts labeled positive for cytoplasmic ZIKV-FLR strain at 4 and 5 dpi, the accumulation of RNA detected +strand by FISH was markedly less (Fig. 4B), and, interestingly, ZIKV was clearly detected in a small population of cells, reminiscent of AXL immunolabeling (Fig. 3B). The − strand labeling by single molecule FISH could not be fully distinguished from autofluorescence, a common problem in primary cell immunofluorescence experiments (Fig. S5). Nevertheless, our qualitative and quantitative results for Zika viral replication are similar to that observed in primary human skin cells^14^, and akin to that observed for hepatitis C in primary trophoblasts^13^ and ZIKV FISH labeling in whole placenta using Affymetrix protocols with an *in vivo* murine model^16^.

**Figure 5:**
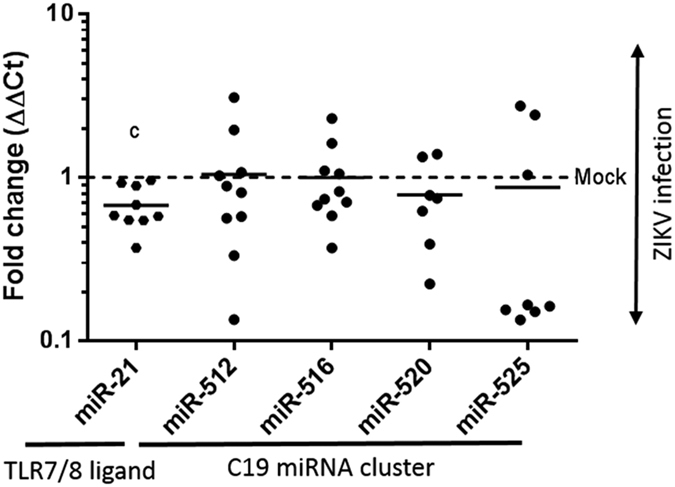
Significant decrease in the level of the TLR7/8 ligand binding miR-21 miRNA, but not C19MC miRNA species following ZIKV infection in primary human trophoblasts. Exosomal RNA was isolated from primary trophoblasts of ten donors following ZIKV infection with 1 × 10^5^ viral copies, or mock infected controls. RNA was then isolated from trophoblast cultures 3–5 days post infection. TaqMan qPCR assays were employed for species-specific miRNA quantification. Significant differences in miRNAs were observed for miR-21 (decreased ~1.5 fold (0.68 ± 0.2 SD), *p* = 0.001), while transcripts of the C19 miRNA cluster were not significantly changed by ZIKV infection. Fold change in miRNA species were calculated by the delta delta Ct method, normalizing first to U6 and then mean delta Ct of mock infected controls. Data was filtered for outliers as designated by a Q of 0.01. Significance was determined using t-tests, with designation of significance annotated by c (c significance *p* = 0.001).

Although miRNA’s primarily function by binding to the 3′-UTR of target mRNAs to achieve post-transcriptional regulation of gene-expression, some miRNA species, including miR-21, ligate the Toll-like immune regulatory receptors (TLRs) to induce inflammatory responses^27^. TLR7 and TLR8 have been recently identified as important sensors of ssRNA from the viral genomes of influenza, vesicular stomatitis virus, and the human and simian immunodeficiency viruses, HIV and SIV^28^. Of particular interest given the neuropathology associated with perinatal ZIKV infection, Yelamanchili *et al*. recently demonstrated that altered expression of miR-21 in extracellular vesicles leads to neurotoxicity via TLR7 signaling during SIV-induced neurological disease^28^. In humans, placental trophoblasts express a distinctive set of primate-specific microRNAs (miRNAs) inclusive of a large cluster on chromosome 19q13 (termed C19MC) as well as the TLR7/8 binding ligand miRNA, miR-21 (recently reviewed in ref. 29). Located along the placental villous interface, the syncytiotrophoblast is reported by other investigators to potentially protect against a number of RNA and DNA perinatal-encountered viral pathogens (with the notable exception of CMV) via certain species of C19MC miRNAs packaged within trophoblast-derived exosomes^30^,^31^. Since C19MC miRNAs and miR-21 may modulate viral replication and inflammatory responses^29^,^30^,^31^,^32^,^33^, we sought to characterize the expression of the miRNAs in primary human trophoblasts following ZIKV infection using qPCR. As shown in Fig. 5, permissive replication of ZIKV (but not DENV, Supplemental Table S3) was accompanied by significant variation in miRNA transcript expression. Specifically, the mean miR-21 was significantly decreased in its expression when infected with ZIKV-FLR (*p* = 0.0014). By comparison, miRNA transcripts of the C19MC cluster (miR-512, miR-516b, miR-520, and miR-525) were unchanged (Fig. 5). This variation in miRNA transcript expression was not observed with DENV inoculation of trophoblast cultures (Supplemental Table S3). Given the postulated role of miRNA species in modulating ZIKV placental infectivity through Type 1 and/or III interferons^11^ we find our significant decrease the TLR binding miR-21 following ZIKV-FLR trophoblast infection of probable interest and likely relevance but acknowledge more studies are needed.

## Discussion

The results of our study indicate that primary human placental trophoblasts (generally assumed to be cytotrophoblasts largely differentiated in culture to non-proliferative syncytiotrophoblasts) are permissive for isolated low passage ZIKV replication, compared with that of DENV. Specifically, replication of ZIKV-FLR was observed both by production of ZIKV RNA, dsRNA and E glycoprotein expression, as well as cytoplasmic localization of virion by FISH. Viral replication did not interfere with syncytiotrophoblast functional differentiation, as evidenced by persistent rises in βhCG production. Moreover, placental trophoblasts express putative ZIKV receptors, and placental miRNAs known to be crucial for ssRNA-ligand sensing TLR7 primate neuropathogenesis (mir-21), are significantly and specifically down-modulated in trophoblasts infected with ZIKV. We speculate that these findings are of potential mechanistic interest to ZIKV pathogenesis in pregnancy^4^,^5^,^6^,^7^,^8^,^9^,^34^,^35^.

Collectively, our data are of likely high significance and importance in deciphering both the clinical manifestations of Zika virus, as well as understanding the underlying biology of viral infectivity and resultant fetal malformations. Because both DENV and ZIKV are flaviviruses which share the same vector (*Aedes* spp), yet have markedly distinct associations with their potential for fetal malformations (ZIKV), or no evidence for fetal nor neonatal affect (DENV), their specific variation in trophoblast permissiveness for replication may be considered to mirror their clinical manifestations. When combined with recent evidence demonstrating fetal neurotrophism^15^, an interesting potential scenario of infectivity emerges. Placental trophoblasts are evidently uniquely positioned to provide both an immunologic and mechanical defensive barrier restricting access of microbes and viruses to the fetus^10^,^11^,^12^,^13^,^14^,^29^,^30^,^31^. However, when placental microbes are either an inherent component of the placental microarchitecture^36^, or actually infect and then replicate in the trophoblast (inclusive of cytotrophoblasts and syncytiotrophoblast), they are then capable of evading innate immune responses. In such an instance, the placenta may be seen as both a reservoir and portal of virus to the fetus. Because the fetus has a patent foramen ovale, the right and left atria in the fetal heart openly communicate and hence provide a conduit for umbilical venous blood directly to the fetal brain. In the case of Zika and other neurotrophic viruses capable of infecting and attenuating hNPC growth and differentiation *in vitro*, along with the release of ZIKV infectious particles^15^, low-level replication of virus in the placental trophoblast and Hoffbauer cell populations^12^,^16^,^17^ would explain the well-documented interval between maternal viremia with resolution in the first or second trimester, and delayed fetal manifestations until the third trimester^4^,^5^,^6^,^7^,^8^,^9^,^37^,^38^. Mlakar *et al*. thoroughly described a case following exposure in Brazil at 13 weeks gestation, without evidence of fetal microcephaly or brain abnormalities throughout the mid trimester until 29 weeks gestation; subsequent autopsy demonstrated concurrence of malformations with intracerebral Zika virus in the fetus^6^. More recently, Driggers *et al*. described a case of a pregnant woman and her fetus being infected at 11 weeks of gestation, with a gradually declining fetal head circumference (but not microcephaly *per se*) through the second trimester^37^. This decline in the fetal head circumference was accompanied by MRI and ultrasound detected brain malformations and persistent maternal viremia with serum positive for ZIKV RNA up until termination of pregnancy at 21 weeks gestation^37^. In this instance, at the time of the delivery the fetal neuronal tissue and the placenta were both positive for ZIKV RNA by PCR, although infectious virions were only isolated from fetal neuronal tissue^37^. Of note, the maternal blood, serum, saliva, urine, plasma, and PBMC were all negative for ZIKV RNA by 11 and 13 days following fetal and placental evacuation from the maternal uterus^37^. Given the rarity of continual fetal neuronal to maternal transmission, these observations are most consistent with the placenta serving as a reservoir and chronic source of detectable virus in the maternal blood and urine.

The primary potential limitation to our study was the overall experimental and donor response variation; however, this has similarly been observed by Quicke *et al*.^12^. Our first two placental trophoblast infectivity experiments (donor 1 and donor 2) demonstrated log-fold viral proliferation (increasing from 1 × 10^5^ to 1 × 10^11^ RNA copies/ml; Fig. 2A), a high level of fold increase not observed in the subsequent donor experiments. There may be multiple factors which led to this discrepancy in viral proliferation. First, the initial two donors were infected with our initial passage of ZIKV FLR strain directly from *A. albopictus* C6/36 cells. Second, there may be donor to donor variation in susceptibility of trophoblasts for ZIKV infectivity and degree of permissivity, as similarly observed by others^12^. Because we aimed to recapitulate the current epidemic, we utilized an experimental design for ZIKV exclusive of latter passages and adapted virus. Ergo, our findings likely reflect the clinical variability and range of both susceptibility and fetal disease^4^,^5^,^6^,^7^,^8^,^9^,^10^,^11^,^12^, with recent retrospective data from the French Polynesia outbreak estimating the occurrence of microcephaly at 1 to 15 in 100 symptomatic gravidae and probable heightened susceptibility with first or early second trimester infection^35^. However, others have shown that a spectrum of perinatal morbidities ranging from fetal growth restriction with and without microcephaly to stillbirth to relatively mild abnormalities in utero-placental function (*e.g.,* shifts in umbilical artery Doppler velocimetry waveforms and oligohydramnios) can occur both proximal and distal to exposure at any gestational age in as great as 1/3 of tested symptomatic gravidae^5^,^6^,^7^,^8^,^9^,^37^. Taken together, these descriptions are consistent with the placenta potentially serving as a reservoir or conduit for later fetal infection, with varying and as yet unexplained susceptibility to clinically evident infections 9,34,35,36,37.

Our findings reported here provide crucial initial evidence that the primary human trophoblasts from uninfected term gestations in a non-endemic population are permissive to contemporary strain ZIKV replication. These observations imply that since the cytotrophoblast progenitor renews throughout the duration of pregnancy, there may be opportunity for persistent low-grade viral replication across gestation in the placenta, which would occur long after initial maternal exposure or viremia has resolved and prior to evidence of observable fetal disease. In such a manner, we surmise the placenta may serve as a relatively silent portal from mother to fetus. Future studies focused on finding *in situ* evidence of active Zika virus replication in the placenta distal from maternal infection, and carefully ascribing gestational age windows of susceptibility in both the placenta and the fetus, will fill in current crucial gaps in our understanding. Similarly, the potential role (or lack thereof) of the trophoblast in triggering an antiviral response following Zika infection *in vivo* needs to be further explored beyond our current miRNA studies reported herein. We further surmise that the capacity for ZIKV to infect placental cells, inclusive of both Hofbauer^12^ and trophoblasts (as shown herein), may additionally explain the relative rapid and widely disseminated spread across the Americas witnessed in the last 15 months. Such spread has previously not been observed with other flaviviruses, and consideration of the role of the placenta and other perinatal portals and reservoirs in arboviral outbreaks deserves future contemplation from both a public health and epidemiologic standpoint.

Despite the limitations to our study, we feel our findings are of likely global importance. It is estimated that the incidence of Zika virus in the general population can be as high as 73% (as occurred on the island of Yap)^38^, rendering risk of primary infection rates in 2015 and 2016 well into the many millions (http://www.cdc.gov/zika/transmission/index.html). Even with a risk of microcephaly being as low as 1% among symptomatic gravidae, the burden to both individual families and society in the face of a burgeoning pandemic is staggering. Time is of the absolute essence as public health officials, scientists and clinicians alike work collaboratively and collectively to decipher the biology and unprecedented cumulative harm of congenital Zika virus infection. It is our hope that these findings, with their notable donor to donor variability but consistency of permissive patterning with a contemporary current strain, will provide avenues and direction for other investigations around the globe. Moreover, the mysteries unraveled by understanding the molecular epidemiology of Zika will undoubtedly enable both advancements in therapy and prevention for the current and future emerging viral epidemics that uniquely affect the developing fetus.

## Methods

### Study design

The intent of this translational study was to determine if primary human placental trophoblasts from uninfected, unexposed, and unrelated donors would be permissive to ZIKV infection. All methods were carried out in accordance with relevant IRB guidelines and regulations, and all experimental protocols were approved by the Baylor College of Medicine Institutional Review Board (IRB). All placental donor subjects were enrolled under Baylor College of Medicine IRB H-26364, and approval for this work was undertaken with IRB approval H-26589. Written informed consent was obtained from all subjects. As part of the consent process, we discussed with participants the potential risks of participation, including the physical risks associated with specimen collection, and the possibility that protected health information or de-identified project data stored in a public repository could be accidentally released. The protocol and consent form described precautions taken to reduce these risks. Additional protections for participants included coding genomic specimens and sequence data, using de-identified medical data, and making efforts to protect subject identification. If a participant withdrew consent after providing specimens, remaining specimens and extracted nucleic acids were to be destroyed; however, any data that were already published in open access databases could not be retracted.

### Placental donor subjects

For comparisons of viral infection in this study, twenty subjects with high integrity placental tissue samples were recruited (Table S2). Inclusion criteria for subjects were term births (where term birth was defined as adequately documented gestational age >37 weeks (by last menstrual period with <20 week gestation sonogram, or by <14 week sonogram with subsequent second trimester sonogram)), and no evidence of major maternal or fetal comorbidity nor anomaly. Subjects were recruited by trained study personnel who approach eligible gravidae at time of admission to labor and delivery. After consent was obtained, clinical metadata was extracted from the electronic medical record or by direct patient enquiry, and redacted data was reserved for future use. For this study, extracted and analyzed clinical metadata included the presence, type, and gestational age(s) of any travel history or risk of clinical infection, the presence or absence of *Streptococcus agalactiae* (Group B strep) on recto-vaginal swab or bacteriuria, and any concern for chorioamnionitis or viremia or active viral infection in the course of the pregnancy. Subjects were not excluded for a history of herpes simplex I or II seropositivity, but were excluded for any evidence of active lesions. All subjects were hepatitis B and C negative, syphilis negative, and HIV negative. The presence of non-infectious asymptomatic bacteriuria and bacterial vaginosis were noted, in addition to the subjects age, race/ethnicity, body-mass-index, diabetic status, and mode and gestational age at delivery.

### Specimen Processing and Trophoblast Isolation and Culture

#### Placental sample collection

All placentae were rigorously collected under strict uniform protocol by perinatal and placental pathology-trained personnel and all were collected within 1 hour of delivery (mean 15 minutes of placental expulsion).

#### Trophoblast isolation and culture

Primary human trophoblasts were isolated from term placentae as previously described^39^,^40^. Briefly, several cotyledons were processed into half inch cubes and further diced with a razor blade. Tissue was digested with 0.25% trypsin and 0.2 mg/mL DNAse I in HBSS to release cells. Trophoblasts were isolated through Percoll gradient centrifugation^41^. Cells which settled in the middle of the gradient were isolated, washed and plated at a density of 2.5 × 10^6^ cells/ well in 24 well plates. Cells were maintained in DMEM/F-12 in 10% FBS with the following antibiotics (Penicillin (1,000 U/L), Streptomycin (0.001% w/v), Gentamycin (0.005% w/v)). Cell were incubated at 37 °C. Media was changed daily until infection and an aliquot of media was saved every 24 hours for βhCG quantification. Purity of the isolation was confirmed by flow cytometry from 3 separate donors by flow cytometry as previously described, and as shown in Supplemental Figure S3^42^.

### Ascertainment of trophoblast differentiation to syncytiotrophoblast with βhCG measures

Cytotrophoblasts are the proliferating cells of the placenta, and further differentiate into villous and extravillous trophoblast cells. Villous trophoblasts will fuse to form multinucleated syncytiotrophoblasts^43^,^44^. These syncytiotrophoblasts line the placental villi and are the physical barrier between maternal and fetal circulation, and control uptake of maternal substances and efflux to the fetal circulation. *In vivo*, as is observed in culture, differentiation from trophoblasts to syncytiotrophoblasts is associated with an increase in secretion of βhCG^39^,^45^. Therefore, differentiation from trophoblasts to syncytiotrophoblasts can be monitored through an increase in βhCG production. Media samples were taken daily from infected and uninfected (mock) cultures from each donor. A commercially available ELISA kit was used to determine βhCG levels according to manufacturer’s protocols (Sigma-Aldrich). A standard curve was determined for each assay using standards provided by the manufacturer. Levels are reported in milli-International Units per milliliter (mIU/mL).

### Ascertainment of trophoblast purity by flow cytometry and cytokine production of infected cultures

Intracellular staining of cytokeratin and vimentin in isolated trophoblasts was performed with the BD Cytofix/Cytoperm kit in accordance to the manufacturer’s protocol. Briefly, Fc receptors were blocked with normal goat serum (Jackson ImmunoResearch) diluted 1/20 in phosphate-buffed saline (PBS). The cells were washed in PBS, fixed and permeabilized with BD Fixation/Permeabilization solution for 20 min at 4 °C, and then washed twice with 1 × BD Perm/Wash buffer. The cells were then incubated with mouse anti-human cytokeratin (Sigma-Aldrich), mouse anti-human vimentin (Sigma-Aldrich) antibodies, or mouse IgG1 isotype control (ThermoFisher Scientific) (10 μg/ml) diluted in 1 × BD Perm/Wash buffer at room temperature for 1 h. After washing twice with 1 × BD Perm/Wash buffer, the cells were incubated in the dark with Alexa Fluor 488 goat anti-mouse IgG (Life Technologies) diluted 1/200 in 1 × BD Perm/Wash™ buffer for 30 min at room temperature. Finally, the cells were washed with twice with 1 × BD Perm/Wash™ buffer, and then analyzed by flow cytometry using the FACSCanto instrument (BD Biosciences) and FlowJo software (Tree Star, Ashland, OR, USA).

Extracellular labeling was performed to trace any potential for contaminating hematopoietic cells in isolated trophoblasts (Fig. S3). After blocking of the Fc receptors with normal goat serum (Jackson ImmunoResearch), cells were washed and incubated on ice for 20 min in the dark with indicated antibodies. Finally, the cells were washed twice with PBS and fixed in PBS containing 1% paraformaldehyde (PFA) for FACS analysis. The following antibodies were used: APC-anti human CD16 (B73.1), PerCP/Cy5.5-anti-human CD25 (M-A251) and PerCP/Cy5.5-anti-human CD56 (B159) was purchased from BD Bioscience. FITC-anti-human-CD15 (HI98), FITC-anti-human CD3 (OKT3), APC-anti-human CD19 (HIB19), and PE-anti-human CD14 (61D3) was obtained from BioLegend. Human peripheral bone marrow mononuclear cells (PBMC) were used as a positive control samples for extracellular labeling with anti-human hematopoietic marker antibodies.

### Isolation of ZIKV-FLR

We have recently described the detailed isolation and characterization of ZIKV FLR strain, including deep sequencing on the Illumina platform^18^. Briefly, under IRB approval, the strain was initially isolated from a 29-year-old nulliparous female who traveled to Barranquilla, Colombia in December 2015 (Baylor College of Medicine IRB H-38650). While symptomatic, testing by quantitative reverse transcriptase polymerase chain reaction (qRT-PCR) for dengue virus (DENV) serotypes 2 and 3^46^, chikungunya virus^47^, and Zika virus (ZIKV)^20^ was performed. Tests for DENV and CHKV were negative, while ZIKV was positive. Details of the methods used to isolate and single passage the virus are provided in ref. 18.

### Phylogenetic Trees Generation

All currently available (*n* = 77; June, 2016 accession) ZIKV complete genome and complete polyprotein CDS assemblies together with Spondweni virus strain SM-6 V-1 were downloaded from the NCBI Nucleotide database (Accessions: KU955595.1, KU955594.1, KU955593.1, KU955592.1, KU955591.1, KU681082.3, KU681081.3, KX247646.1, KX185891.1, KU866423.1, KX056898.1, KJ776791.1, KX280026.1, KX197192.1, KU744693.1, KX117076.1, KU509998.3, KU963796.1, KU321639.1, KU991811.1, KU870645.1, KU926310.1, KU926309.1, KU922960.1, KU922923.1, KU820898.1, KU740184.2, KU853013.1, KU853012.1, KU729217.2, KU729218.1, KU761564.1, KU720415.1, KU497555.1, KU707826.1, KU527068.1, NC_012532.1, KU647676.1, KU501217.1, KU501216.1, KU501215.1, KU365780.1, KU365779.1, KU365778.1, KU365777.1, KU312312.1, KF268950.1, KF268949.1, KF268948.1, LC002520.1, KF383119.1, KF383118.1, KF383117.1, KF383116.1, KF383115.1, AY632535.2, EU545988.1, KU758877.1, KX247632.1, KX262887.1, KX253996.1, KU820897.2, KX087101.2, KX198135.1, KX198134.1, KX156776.1, KX156775.1, KX156774.1, KU937936.1, KX087102.1, KX051563.1, KU955590.1, KU955589.1, KU963574.1, KU963573.1, KU820899.2, DQ859059.1, DQ859064.1). Sequences were aligned using MAFFT v7.0 (Katoh)^23^ with the accurate (L-INS-i) setting. PAUP* v4.0 (Swofford)^24^ automated model selection was used to identify optimal likelihood parameters based on the best AICc (GTR + I + G; nst = 6 rclass = (abcdef) rmatrix = (1.0321439 6.5676849 1.547758 0.49119007 20.584045)basefreq = (0.27644007 0.23772344 0.28277434) rates = gamma shape = 1.8552617 pinv = 0.49424577). A Neighbor Joining tree was constructed and the tree was visualized using FigTree v1.4.2 (Rambaut; http://tree.bio.ed.ac.uk/software/figtree/). Leaf nodes were labelled as country_host_year of collection based on availability of this information at NCBI or in associated publications.

### ZIKV FLR Propagation and Quantification

#### Propagation

Zika strain FLR was propagated once in C6/36 cells (harvested day 14 post infection) and aliquots were stored in 30% FBS, at −70 °C. Dengue serotype 2 virus, strain K0049 was a stock that had been passaged 3 times.

#### Real-time or quantitative viral RT-PCR

Viral RNA was extracted from ZIKV C6/36 cell culture supernatants and DENV stocks by using TRIzol^®^ LS reagent according to the manufacturer’s instructions (Thermo Fisher Scientific). The assay is based on the amplification of a ZIKV envelope gene region^20^, and a DENV capsid region^46^, using ZIKV and DENV-2 specific primers and probes, listed in Table S1. The reactions were performed using the TaqMan^®^ Fast Virus 1-Step Master Mix kit (Applied Biosystems). The concentration of both ZIKV and DENV viral RNA (copies/milliliter) was estimated by using the standard curve generated from DENV2 transcripts^46^. This standard curve was later compared to ZIKV RNA measured by spectrophotometry, to confirm their direct correlation.

#### ZIKV and DENV Infection of Primary Human Trophoblasts

A total of 20 donors placental trophoblasts were tested for ZIKV FLR strain permissive replication, with 14 of these used for comparisons with dengue virus replication, and 2 for cytokine comparisons between Zika-infected and uninfected cells of the same donor. On day 0 of post isolation, purified primary trophoblasts were seeded onto each well of a 24 well-plate at either 1 × 10^5^ or 1 × 10^6^ cells. Medium was changed every day prior to infection. After 2 to 5 days of plating (post isolation days 2, 3, 4 or 5), medium was removed and cells were incubated/infected with 1 × 10^5^ RNA copies of ZIKV (10 TCID) and DENV (100 TCID) for 1 h at 37 °C. Inoculum was replaced with fresh F-12 (DMEM/F12) culture medium containing 10% FBS and antibiotics, and the cells were maintained at 37 °C and 5% CO_2_. Fifty μL of culture supernatant was taken from each of two wells (biological duplicates) at 24 hour interval times post infection (hours or days post-infection, dpi), and viral RNA was extracted and measured as described above employing qRT-PCR.

#### Qualitative evidence of infection using immunofluorescent antibody labeling

1 × 10^5^ PHT cells were seeded onto chamber slides (Nunc, Lab-Tek II) 24 h before infection with ZIKV or UV inactivated ZIKV. At 5 days post infection, cells were fixed in ice-cold acetone for 20 min. Cells were washed with PBS and incubated with blocking solution (1%BSA in PBS-T) for 30 min at 37 °C, drained access amount of blocking solution and incubated with anti-Flavivirus monoclonal antibody 4G2 (EMD Millipore) or J2 anti-ds RNA monoclonal antibody for 2 h at 37 °C. Cells were washed with PBS-T and incubated for 1 h at 37 °C with Alexa flour-488 or Alexa flour-568 conjugated anti-mouse IgG antibodies (Life Technologies). Cells were washed with PBS or PBS-T three times and mounted with ProlongGold^®^ antifade reagent with DAPI (cell signaling). Images were analyzed under a fluorescence microscope (Fig. 1).

#### UV-inactivation of ZIKV

ZIKV cell culture supernatants were exposed to 1.8 jules/cm^2^ of UV illumination according to manufacturer’s instructions (Stratagene UV crosslinker) and used to infect primary human trophobalsts.

#### Cytokine secretion assay

Cytokine production by infected and uninfected trophoblasts were assessed via luminex assay, using kits to detect 12 human cytokines: IFNγ, IL-10, sCD40L, IL-1RA, IL-2, IL-4, IL-6, IL-8, MCP-1, RANTES, TNFα, and VEGF. Supernatant samples (25 μL) were collected daily and stored at −70 C. Luminex assay was performed using the Milliplex Human Cytokine/Chemokine Magnetic Bead Panel (Millipore) per manufacturer’s instructions. To improve assay sensitivity, samples were incubated with the magnetic beads overnight at 4 °C. Samples were run on a MAGPIX instrument and analyzed with xPONENT software. The results from two donors are shown in Fig. S4.

### Statistical Analysis

Results were analyzed using GraphPad Prism (v. 7). Quantities of viral RNA produced by trophoblasts were compared by student’s two-tailed t tests, with significances noted at *p =>* 0.05 (*), >0.01 (**), >0.001 (***), >0.0001 (****). Cytokine values were compared by student’s t test with correction for multiple comparisons using the Holm-Sidak method.

### Single molecule RNA fluorescence *in situ* hybridization (FISH)

Single molecule RNA FISH assays were carried out using a custom designed oligonucleotide probe set (Stellaris^®^, LGC Biosearch Technologies). The probe set was designed against the KU365780.1 nts 125–8011 (designed to detect the positive polyprotein ZIKV RNA strand *in situ*), spanning the consensus region. A negative strand probe set was also designed against reverse complement of KU365780.1, nts c8011–125, spanning a second consensus region. 47 (+strand) oligonucleotides or 48 (−strand) oligonucleotides were selected with minimal mismatch to the consensus of AY632535.2, DQ859059.1, EU545988.1, KF268948.1, KF268949.1, KF268950.1, KF383115.1, KF383116.1, KF383117.1, KF383118.1, KF383119.1, KJ776791.1, KU312312.1, KU321639.1, KU365777.1, KU365778.1, KU365779.1, KU365780.1, LC002520.1, and NC_012532.1. Each oligo to the positive strand was co-synthetically labeled with one Quasar^®^ 570 dye (a cyanine 3 analog) at the 3′- end; the negative viral strand probe set was post-synthetically 3′-labeled with ATTO 647 N, and further purified by HPLC to remove unreacted dye. Probes were concomitantly visualized with cell nuclei that were stained with the DNA intercalator 4′,6-diamidino-2-phenylindole (DAPI).

RNA FISH was carried using hybridization and wash buffers from LGC Biosearch Technologies as follows. 10^5^–10^6^ primary human trophoblasts or Vero cells were seeded on poly-L-lysine-coated coverslips, or on 24 well plates with optical plastic bottom (IBIDI). Cells were maintained in culture at 37 °C and 5% CO_2_ with (DMEM/F12, 5% FBS and 1% penicillin) for up to 5 days to syncytialize, and then were infected with 1 × 10^5^ RNA copies of ZIKV (10 TCID) for 1 h at 37 °C. The cells were then maintained in new media for up to 5 days post infection. Cells were fixed in 4% purified formaldehyde (Electron Microscopy Sciences) in RNase-free phosphate-buffered saline for 30 min on ice and then permeabilized with 70% ethanol in RNase-free water at 4 °C. At all points in the fixation and permeabilization steps, cells were monitored by phase microscopy. Cells were washed in 1 ml of wash buffer (2x SSC (Ambion) plus 10% formamide) followed by overnight hybridization at 37 °C with FISH probes in hybridization buffer (LGC Biosearch Technologies) followed by one change of wash buffer for 30 min at 37 °C, and then DNA staining with DAPI (1 μg/ml) for 10 min at 37 °C. Finally, cells were washed in 2x SCC buffer, and coverslips were mounted in Vectashield anti-fade (VectorLabs). Experiments in multiwell plates were maintained in 2x SSC buffer and immediately imaged. Imaging was performed on a GE Healthcare DV Live deconvolution microscope using an Olympus 60x/1.42 PlanApo objective. Z-stacks were collected (0.3 μm steps) for several fields per sample. To account for autofluorescence in the trophoblast samples, the FITC channel was collected for comparison to ensure true signal (Fig. 4 and S7). Image z-stacks were deconvolved using the restorative deconvolution algorithm in the SoftWoRx™ software and max-intensity projected. For reduction of autofluorecence, images were processed in Photoshop, subtracting the FITC channel image from the probe channel.

### Assessment of Putative ZIKV Viral Receptor Expression by RT-PCR and Immunofluorescence

*RT PCR.* Trophoblasts were isolated from healthy donors as described above and cultured *in vitro* for 5 days with daily media changes of DMEM/F-12 (Gibco) with 10% FBS (Atlanta Biologicals). Cells were then washed with PBS and saved by scraping adherent cells in RNA*later* (ThermoFisher) and subsequently stored at −80 °C for later extraction. For control, cryopreserved human hepatocytes or healthy human whole liver were used, alongside freshly isolated human buffy coat cells (1 × 10^6^) or the cell line A549 cells. Isolated hepatocytes were purchased from CellzDirect (Life Technologies) and whole liver pieces were procured upon liver resections. Total RNA was then extracted from thawed cells using the Nucleospin RNA isolation kit (Macherey-Nagel, Duren, Germany) according to manufacturer’s instructions. The quality and yield of the RNA isolation was assessed by Nanodrop, and 1 μg cDNA libraries were prepared using High Capacity cDNA Reverse Transcription Kit (Applied Biosystems) according to instructions. Levels of putative receptors were then assessed by RT-PCR using GoTaq DNA Polymerase (Promega, Madison, WI) in 25 ng reactions at 57 °C for all primers. The primers for DC-SIGN, L-SIGN, TYRO3, Axl, and TIM-1 were designed against NCBI RefSeqs with the assistance of Primer3 software^48^, and verified using UCSC Genome bowser *in silico* PCR (http://genome.ucsc.edu)^49^. To account for the many splice variants and sequence similarity of the highly homologous DC-SIGN and L-SIGN transcripts, primers were designed in regions common to the splice variants but unique to the respective transcripts with the assistance of SpliceCenter software (*In Silico* Solutions, Falls Church, VA)^50^. Major bands were found at 707 bp (DC-SIGN), and 229 bp (L-SIGN), and 166 bp (L-SIGN), corresponding to RefSeqs DC-SIGN (CD209) transcript variant 1, L-SIGN (CLEC4M) transcript variants 1, 8, 9 and 10, and L-SIGN (CLEC4M) transcript variants 7 and 11 respectively. These band sizes were therefore used to evaluate transcriptional changes of CD-SIGN and L-SIGN. For all primer sets, at least one primer was designed to span an exon junction to avoid genomic bands. Assessment of transcription was made by comparison against a previously validated GAPDH loading control^51^. Primer sequences are listed in Table S1.

#### Immunolabeling and imaging of cell surface receptors

Putative viral receptors were assessed for expression by immunofluorescence. Monoclonal mouse antibodies against Human DC-SIGN/L-SIGN (clone 120612), TYRO3 (clone 96201), TIM-1 (clone 219211) and goat polyclonal antibody for human Axl were obtained from R&D Systems (Minneapolis, MN); Alexa Fluor^®^ 647 labeled secondary antibodies were from Thermo Fisher (Waltham, MA). As with FISH (above), 10^5^–10^6^ primary human trophoblasts were seeded on 24 well plates with optical plastic bottom (IBIDI), maintained for 5 days, and then infected with 1 × 10^5^ RNA copies of ZIKV (10 TCID) for 1 h at 37 °C. Cells were then fixed 5 days post infection with 4% formaldehyde for 30 minutes on ice. Wells were washed 1x with PBS, quenched with 0.1 M NH4Cl and permeabilized with triton X100. After 1 hr of blocking in blotto (5% milk in TBST), cells were incubated with primary antibodies (1:500) overnight at 4 °C. The cells were then washed 4x for 5 min in blotto, and incubated for 30 minutes at room temperature with 1:1000 dilution of the secondary antibodies. After 4x washes in TBST, cells were then counterstained with DAPI (1 μg/ml) for 2 min. Wells were kept in PBS and immediately imaged. Imaging was performed on a GE Healthcare DV Live deconvolution microscope using an Olympus 20x/0.75 UPlanApo objective in 0.7 μm z-stacks, deconvolved and max-intensity projected.

### miRNA Analysis

ZIKV infected or mock trophoblast cells were collected following culture experiments in RNAlater (Ambion) and stored at −80 °C until extraction. Total RNA was extracted using mirVana miRNA Isolation Kit (Ambion) according to manufacturer instructions. Extracted RNA yield was assessed by Nanodrop (ThermoFIsher Scientific). Levels of specific miRNAs were quantified using TaqMan qPCR assay Kits (ThermoFIsher Scientific) for hsa-miR-21–5p, hsa-miR-512–3p, hsa-miR-516b-5p, hsa-miR-520a-5p, and hsa-miR-525–5p with U6 snRNA as an endogenous house-keeping control. Specific assay were 397, 1823, 1150, 1168, 1174, and 1973 for hsa-miR-21–5p, hsa-miR-512–3p, hsa-miR-516b-5p, hsa-miR-520a-5p, hsa-miR-525–5, and U6 respectively. Template was prepared from 20 ng extracted RNA using assay specific primers for each microRNA measured using TaqMan MicroRNA Reverse Transcription Kit (ThermoFisher Scientific). Quantitative real time PCR reactions were prepared using Universal PCR Master Mix (Applied Biosystems by Life Technologies) as per manufacturer’s instructions, and miRNA levels assessed on using a StepOnePlus platform (Applied Biosystems). Changing levels were calculated by delta delta Ct method by first normalizing to an U6 endogenous control by subtraction, and then comparing to mock biological controls. Fold change was calculated from the delta delta Ct comparison. Differences were determined by paired t-tests to account for donor to donor variation. All statistics were performed with Prism software (GraphPad, v6.01, La Jolla, CA).

## Additional Information

**How to cite this article**: Aagaard, K. M. *et al*. Primary Human Placental Trophoblasts are Permissive for Zika Virus (ZIKV) Replication. *Sci. Rep.* **7**, 41389; doi: 10.1038/srep41389 (2017).

**Publisher's note:** Springer Nature remains neutral with regard to jurisdictional claims in published maps and institutional affiliations.

## Supplementary Material

Supplementary Data
