# Supplementary material for: Primary Human Placental Trophoblasts are Permissive for Zika Virus (ZIKV) Replication
Source: Sci Rep. 2017 Jan 27;7:41389. doi: 10.1038/srep41389 (PMC5269613; doi:10.1038/srep41389)
Supplement: Supplementary Data [file srep41389-s1.pdf]

Supplemental Information for:

Primary Human Placental Trophoblasts are Permissive for Zika Virus (ZIKV) Replication

Authors: *Kjersti M. Aagaard[&,1,2,3,4], Anismrita Lahon[5], Melissa A. Suter[1], Ravi P. Arya[5], Maxim D. Seferovic[1], Megan B. Vogt[5,6], Min Hu[1], Fabio Stossi[3], Michael A. Mancini[3], R. Alan Harris[1,2], Maike Kahr[1], Catherine Eppes[1], Martha Rac[1], Michael A. Belfort[1], Chun Shik Park[7], Daniel Lacorazza[7], and Rebecca Rico-Hesse[5].*

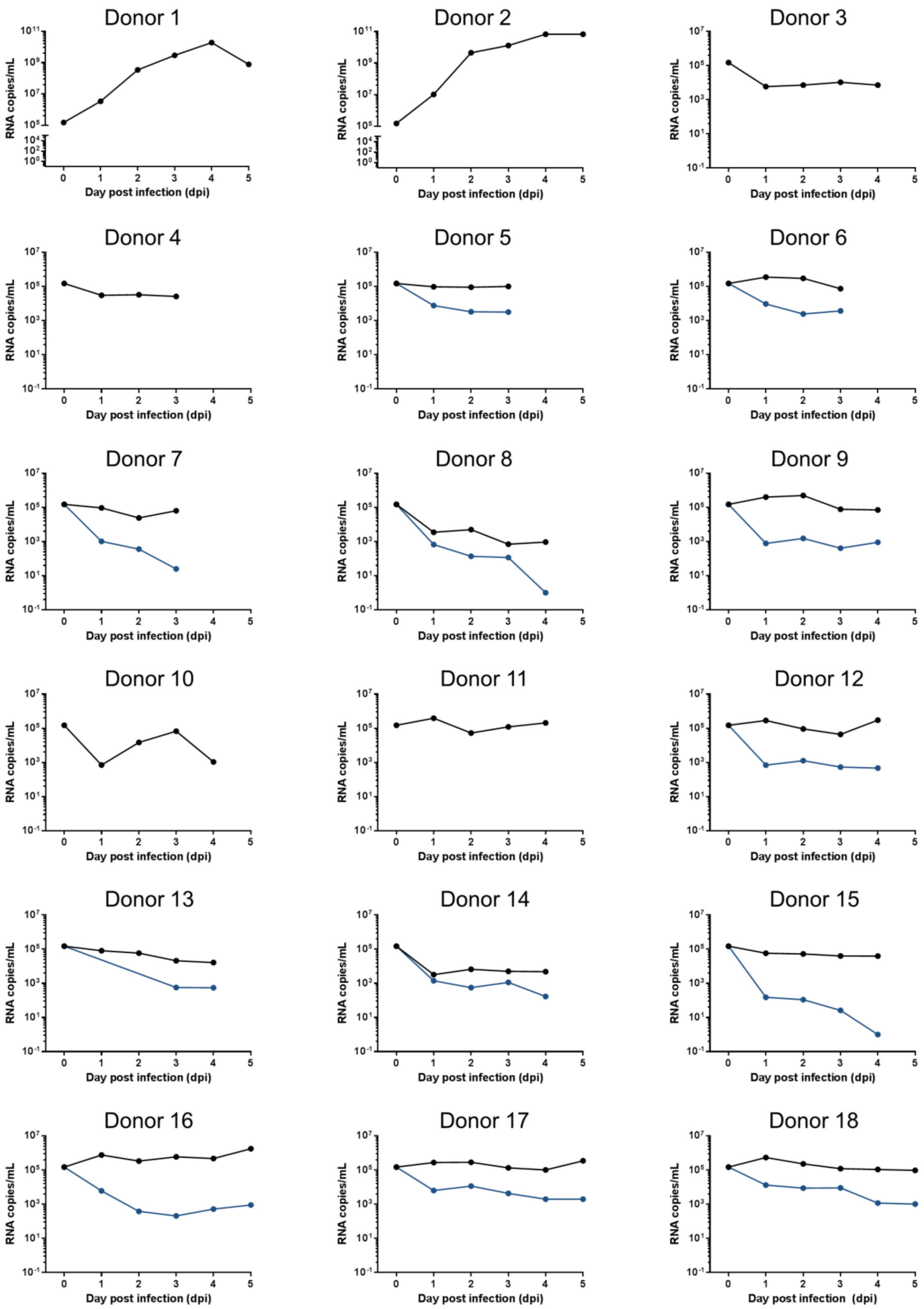

Fig. S1. Detection of ZIKV RNA is observed up to 5 days post inoculation of unrelated, non-endemic primary human trophoblast donor cultures. Zika (ZIKV; black) and dengue (DENV; blue) viral RNA

quantification curves infected donors measured. Individually plotted data shown here were combined to generate Fig 2. Donors 3 and 4 were infected on day 2 post isolation, Donors 5, 6, 16, & 18 were 3 days post isolation, donors 1, 7-12, & 17 were 4 days post isolation, and donors 2 & 13-15 were 5 days post isolation. Measures of ZIKV and DENV were obtained both at inoculation (d0 dpi) and multiple time points thereafter (d1 through d5 dpi, x-axis).

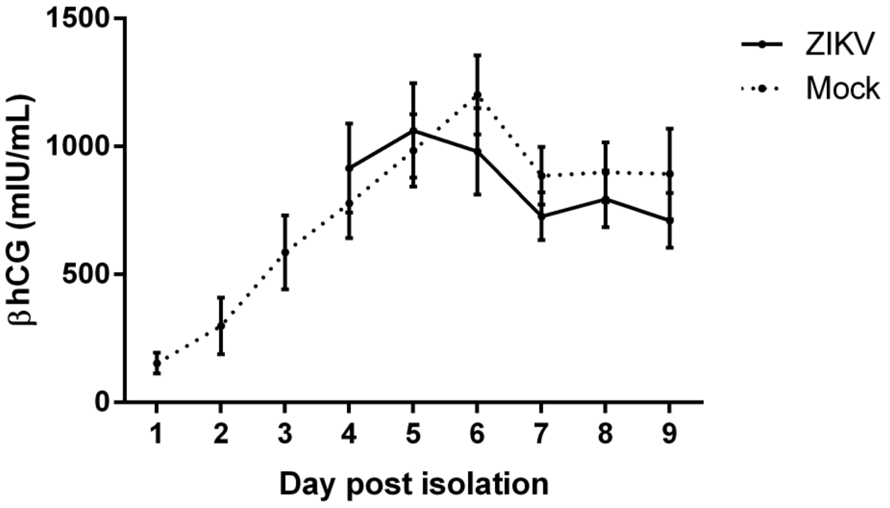

Fig. S2. βhCG production by differentiated primary trophoblasts is unchanged with ZIKV-FLR infection. Quantitative βhCG measurements were obtained from culture media, with aliquot sampling every 24 hours. A commercially available ELISA kit was used to determine βhCG levels according to manufacturer's protocols (Sigma-Aldrich). A standard curve was determined for each assay using standards provided by the manufacturer. Levels are reported in milli-International Units per milliliter (mIU/mL). Determination of βhCG production was performed in over half of all placental donor subjects (*n*=11 of a total of 20 subjects) at 24 hour time point. No difference was observed with ZIKV infection (solid lines) compared with mock infection controls (dashed lines).

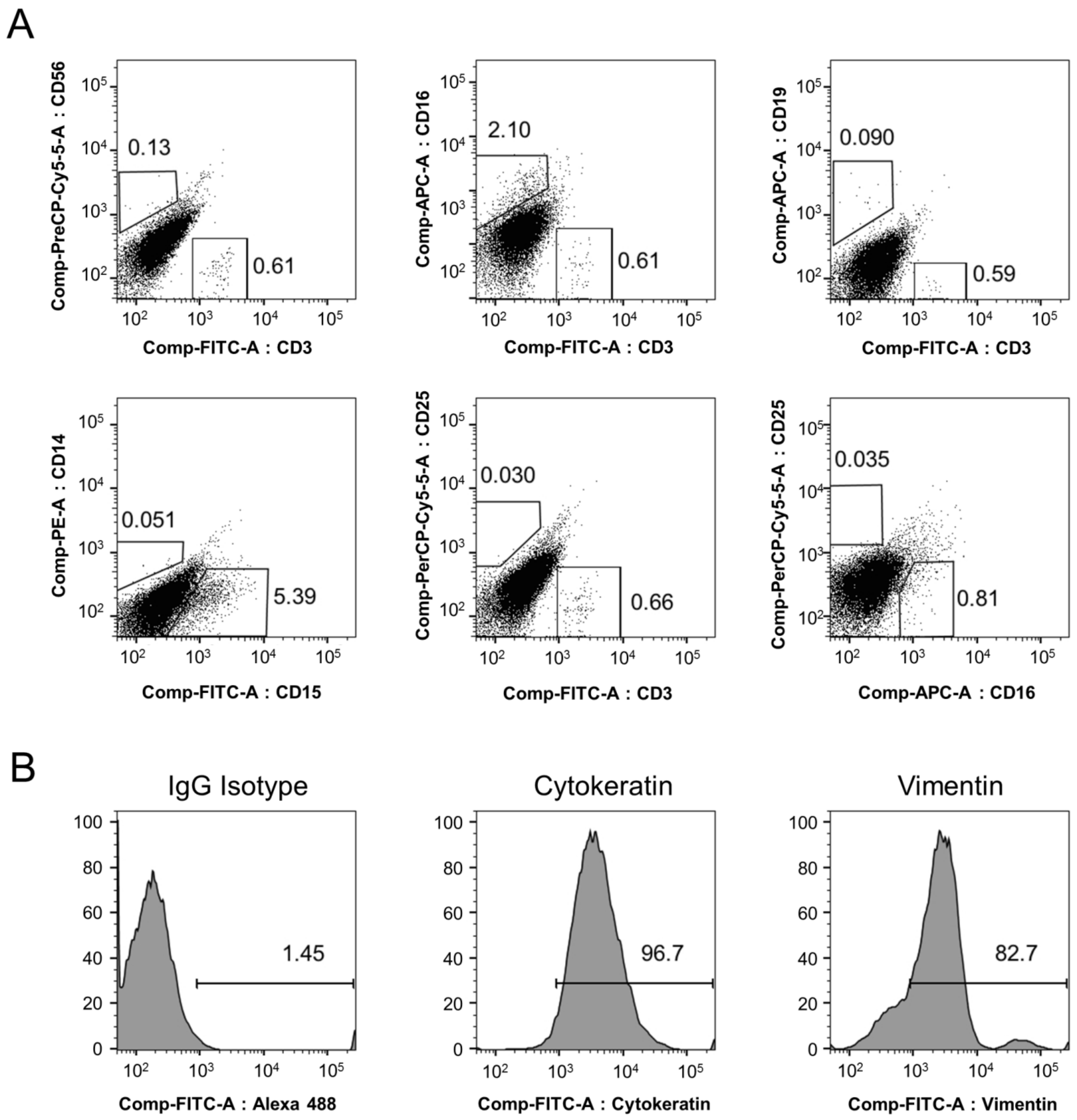

Fig. S3. Flow cytometry analysis. (A) Flow cytometric analysis of human hematopoietic markers CD3, CD14, CD15, CD16, CD19, CD25, and CD56 in the isolated trophoblasts. Numbers represent the percentage of cells in an outlined gate of representative dot plots. (B) Intracellular staining of cytokeratin and vimentin in the isolated trophoblast samples. As a negative control, mouse IgG1 isotype was stained. Numbers represent the percentage of cells expressing cytokeratin or vimentin in the isolated trophoblast samples. Findings of FACS analysis shown here represent 3 separate donor labeling studies.

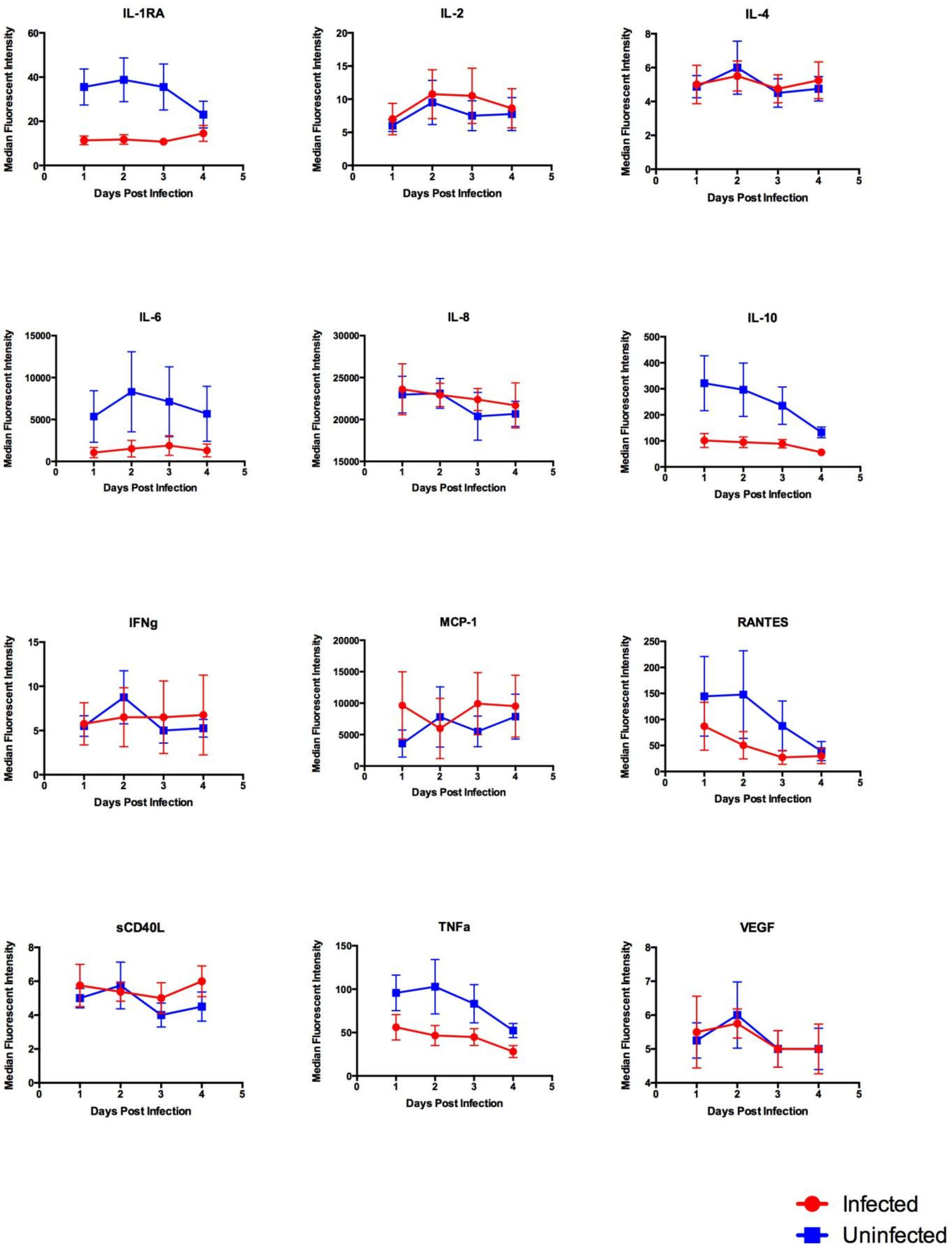
IL-1RA
IL-2
IL-4
IL-6
IL-8
IL-10
IFNg
MCP-1
RANTES
sCD40L
TNFa
VEGF
Median Fluorescent Intensity
Days Post Infection
Infected
Uninfected

Fig. S4. Time course of secretion of 12 different cytokines, by ZIKV FLR infected and uninfected primary human trophoblasts, from two different donors (12 and 13). Cytokine production by infected and uninfected trophoblasts were assessed via luminex assay, using kits to detect 12 human cytokines: IFNγ, IL-10, sCD40L, IL-1RA, IL-2, IL-4, IL-6, IL-8, MCP-1, RANTES, TNFα, and VEGF. Supernatant samples (25 μL) were collected daily and stored at -70°C. Luminex assay was performed using the Milliplex Human Cytokine/Chemokine Magnetic Bead Panel (Millipore) per manufacturer's instructions. To improve assay sensitivity, samples were incubated with the magnetic beads overnight at 4°C. Samples were run on a MAGPIX instrument and analyzed with xPONENT software.

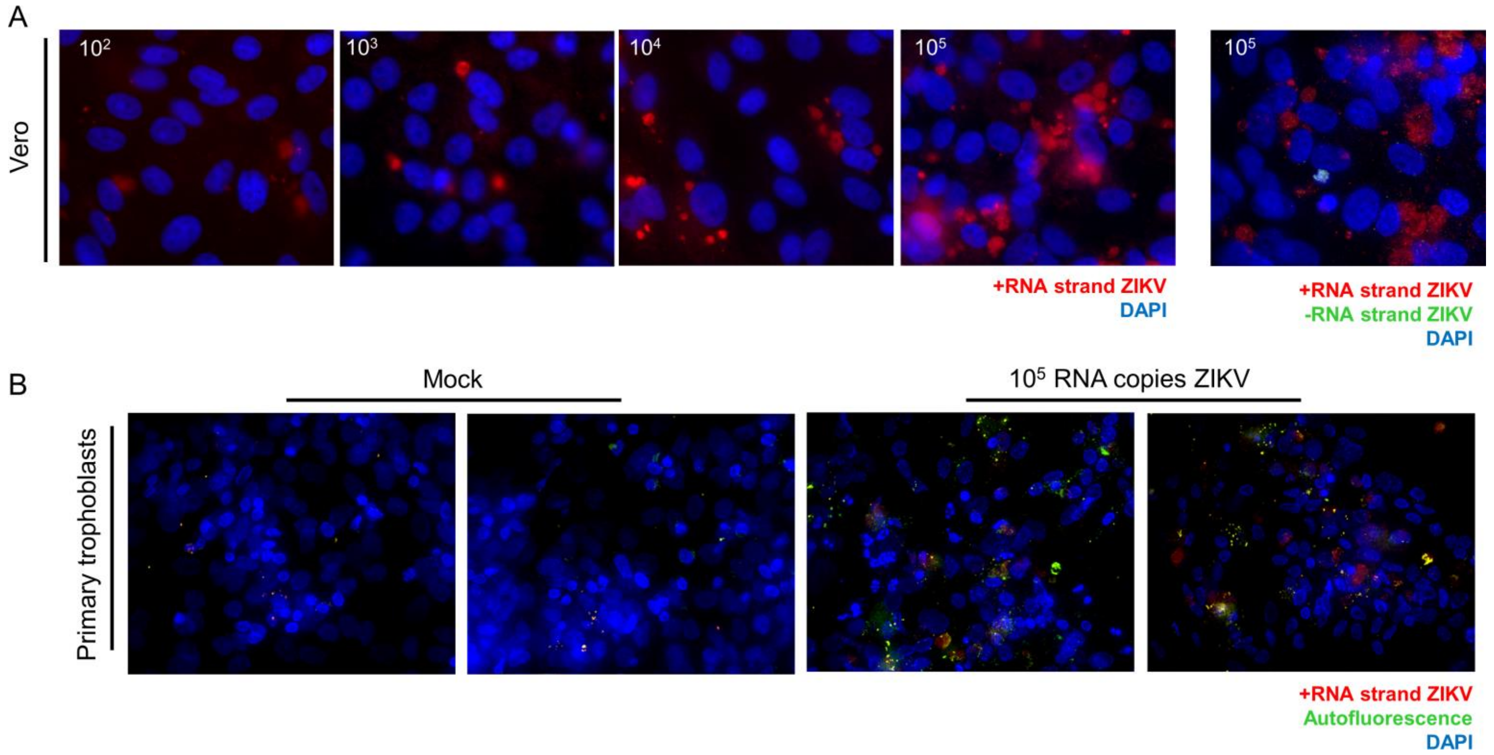

Fig. S5. Viral replication in Vero and primary human placental trophoblasts by single molecule RNA FISH. (A) Vero cells infected with a titration of ZIKV-FLR strain from 10^2-10^5 RNA copies were probed with specific fluorescently-labeled oligo panels to ZIKV RNA positive strand (red) at 5 dpi as detailed in Fig 4. Negative strand probes (green) revealed signal in some clusters for all concentrations. (B) Primary human placental trophoblasts as in figure 4 infected 4 days post isolation with ZIKV FLR strain at 1 x 10^5 RNA copies, and fixed at 5 dpi. Select 60X magnifications from three separate experiments show distinct ZIKV +RNA viral strand signal (red) separate from background FITC channel (green).

qPCR and RT PCR Primer and Probe Sets				
**Target**	**Primer /probe**	**Genome position**	**Sequence (5'- 3')**	**Reference**
ZIKV	ZIKV 1086	1086–1102	CCGCTGCCCAACACAAG	(*10*)
	ZIKV 1107-FAM	1107–1137	AGCCTACCTTGACAAGCAGTCAGACACTCAA	
	ZIKV 1162c	1162-1139	CCACTAACGTTCTTTTGCAGACAT	
DENV	DENV2/141	141-160	GCTGAAACGCGAGAGAAACC	(*8*)
	DENV2/177-FAM	177-209	TGTCGACTGTTTCTCTAAGAGTGAACCTTACGA	
	DENV2/234	212-234	TCCCTGCTCCTGGTIATTTTGAC	
DC-SIGN	DCSIGNFwd		AGTCAGGAACAATCCAGGCA	
	DCSIGNRev		AGGAAGTTCTGCTCCTCAGC	
L-SIGN	LSIGNFwd		TGGGCCTCCTGGAAGAAGAT	
	LSIGNRev		GCGTCTTGCTCGGATTGTTC	
TYRO3	TYRO3Fwd		CACGGTAGAAGGTGTGCCAT	
	TYRO3Rev		CCTGAGGCTGTAGTTGGTGG	
AXL	AXLFwd		GCGTCTTGCTCGGATTGTTC	
	AXLRev		CCCCGAAGGACCACACATC	
TIM-1	TIM1Fwd		AACCATGAACCAGTAGCCACT	
	TIM1Rev		AACCATGAACCAGTAGCCACT	
GAPDH	GAPDHFwd		GCACCACCAACTGCTTAGCA	(*19*)
	GAPDHRev		GTCTTCTGGGTGGCAGTGATG	

Table S1. List of q/RT-PCR primer/probes for ZIKV, DENV2, and their putative receptors. Reagents for miRNA experimentation are provided in Supplemental Methods text.

Characteristics of Placental Donors										
**Subject Number**	**MOD**	**GA (wks)**	**Race**	**Ethnicity**	**Comorbidities**	**Age**	**PP BMI**	**Medications in Pregnancy**	**Infant Sex**	**Post Isolation DOI**
**1**	V	39 0/7	White	Hispanic	Gestational Diabetes, type A2	29	29.6	PNV, insulin	M	5
**2**	V	39 5/7	White	Hispanic	None	32	27.3	PNV	F	4
**3**	V	38 6/7	White	Hispanic	Anxiety	20	23.3	PNV, citalopram	M	2
4	V	39 1/7	White	Hispanic	History of PRE in previous pregnancy	34	20.2	PNV, aspirin	F	2
**5**	V	39 4/7	Black	Non-Hispanic	None	26	NR	PNV	F	3
**6**	V	37 2/7	White	Hispanic	None	27	32.9	PNV	F	3
**7**	V	38 3/7	White	Hispanic	None	25	25.9	PNV	F	4
**8**	C	39 0/7	White	Hispanic	Asthma, Remote history of HSV II seropositive without lesions, morbid obesity	30	38.4	PNV, albuterol as needed	F	4
**9**	V	39 3/7	White	Hispanic	PRE without severe features	19	25.6	PNV	M	4
**10**	V	38 3/7	White	Hispanic	HSV II seropositive, without lesions	33	23.4	PNV, prescribed acyclovir prophylaxis at 36 weeks	F	4
**11**	C	39 1/7	White	Hispanic	Oligohydramnios	27	NR	PNV	F	1,2,3,4 & 5
**12**	C	39 2/7	White	Hispanic	None	32	27.4	PNV	F	4&5
**13**	V	41 6/7	White	Hispanic	N/A	24	26.5	PNV	M	5
**14**	V	37 1/7	White	Hispanic	Gestational Diabetes, type A1	23	39.3	PNV	F	5
**15**	C	38 1/7	White	Hispanic	Anemia	24	23.9	PNV, ferrous sulfate	F	5
**16**	C	37 2/7	Black	Non-Hispanic	PRE without severe features	30	29.9	PNV	M	3
**17**	C	39 1/7	White	Hispanic	None	26	24.0	PNV	M	4
**18**	V	37 6/7	NR	Hispanic	None	40	27.5	PNV	M	3
**19**	C	40 4/7	White	Hispanic	None	20	27.2	PNV	M	4
**20**	V	37 0/7	White	Hispanic	None	25	30.1	PNV	F	4

Table S2: Characteristics of the subjects enrolled in this study. ID: identification; MOD: mode of delivery; V: vaginal; C: cesarean; GA: gestational age at delivery reported as weeks and days of gestation; GMDA2: gestational diabetes mellitus A2; Hx: history; PRE: preeclampsia, IUFD: intrauterine fetal demise; PP BMI: pre-pregnancy body mass index; Post Isolation DOI: "day of infection," specifically, what day post trophoblast isolation cells (day of collection and isolation defined as day 0) were infected with mock, ZIKV or DENV; PNV: prenatal vitamins; F: female; M: male.

**miRNA Expression Following DENV Inoculation in Primary Human Trophoblasts**			
**miRNA**	**Fold change**	**Standard Deviation**	***p* value**
miR21	2.6	3.9	0.25
miR512	7.8	12.3	0.16
miR516	5.9	7.0	0.07
miR520	7.0	7.7	0.16
miR525	1.013	1.3	0.98

Table S3: miRNA expression in primary human trophoblasts is not significantly varied following DENV inoculation. Exosomal RNA was isolated from primary trophoblasts as detailed in Figure 5. RNA was then isolated from trophoblast cultures 3-5 days post infection. TaqMan qPCR assays were employed for species-specific miRNA quantification. No significant differences in miRNAs were observed for miRNA transcripts following DENV inoculation when compared to mock-infected controls. Fold change in miRNA species were calculated by the delta delta Ct method, normalizing first to U6 and then mean delta Ct of mock infected controls. Significance was determined using t-tests, with a p value of <0.05 denoting significance.
